# Genomic Characterization of Mycoplasma arginini Isolated from a Housefly on a Dairy Farm and Comparison with Isolates from Bovine Milk and Lung Tissue

**DOI:** 10.1128/spectrum.03010-22

**Published:** 2023-05-18

**Authors:** G. Gioia, M. Severgnini, P. Cremonesi, B. Castiglioni, J. Freeman, A. Sipka, C. Santisteban, M. Wieland, V. Alanis Gallardo, J. G. Scott, P. Moroni, M. F. Addis

**Affiliations:** a Quality Milk Production Services, Animal Health Diagnostic Center, Cornell University, Ithaca, New York, USA; b Institute of Biomedical Technologies, National Research Council, Segrate, Milan, Italy; c Institute of Agricultural Biology and Biotechnology, National Research Council, Lodi, Italy; d Department of Entomology, College of Agriculture and Life Sciences, Cornell University, Ithaca, New York, USA; e Departamento de Medicina Preventiva y Salud Pública, Facultad de Medicina Veterinaria y Zootecnia, Universidad Nacional Autónoma de México, Ciudad Universitaria, Mexico City, Mexico; f Dipartimento di Medicina Veterinaria e Scienze Animali, Università degli Studi di Milano, Lodi, Italy; g Laboratorio di Malattie Infettive degli Animali-MiLab, University of Milan, Lodi, Italy; Institute of Parasitology, Biology Centre, ASCR

**Keywords:** *Mycoplasma*, housefly, dairy farm, mastitis, milk, cow

## Abstract

*Mycoplasma mastitis* can be highly contagious, unresponsive to treatment, and cause severe economic problems in affected herds. Notable routes of *Mycoplasma* spp. transmissions are contaminated milking equipment and animal contact through respiratory secretions. Only a few studies report the environment as a possible source of infection. Our group studied the presence of pathogens in houseflies (Musca domestica) in a New York State dairy in the United States. Among others, a *Mycoplasma* spp. was found in the gut of a housefly captured in the sick pen and identified as *M. arginini*. Here, we characterized its genome and investigated its relatedness with eight isolates from milk, one isolate from lung tissue collected in the same dairy, and five other dairies in New York State. We applied whole-genome sequencing and phylogenetic analysis based on the sequences of the 16S rRNA gene and 76 conserved proteins. We also assessed an *in silico* virulence profile by considering a panel of 94 putative virulence genes. As a result of the genome analysis, the housefly *M. arginini* isolate was highly similar to the milk isolates; interestingly, the similarity was highest with *M. arginini* isolated from milk on the same dairy farm where the housefly was captured. The housefly and milk *M. arginini* isolates possessed 54 of the 94 pathogenicity genes considered. Our data support the hypothesis that houseflies are carriers of *Mycoplasma* spp. and can be considered within the possible roots of environmental transmission of infection in dairy cows. Nevertheless, *M. arginini* pathogenicity will need to be investigated with dedicated studies.

**IMPORTANCE** It is critical to control the spread of bovine mastitis caused by *Mycoplasma* spp., as this disease can be highly contagious and have a severe economic impact on affected dairies. A better understanding of possible transmission routes is crucial for infection control and prevention. Based on our data, the composite milk isolates are genetically similar to the housefly isolate. This provides evidence that the same *Mycoplasma* species found in milk and associated with mastitis can also be isolated from houseflies captured in the dairy environment.

## INTRODUCTION

*Mycoplasma* spp. are a significant concern for dairy farms, being associated with several diseases in cattle, including mastitis, pneumonia, otitis, arthritis, and reproductive disorders (1). Herds in the United States and many other countries have a zero-tolerance policy for cows with mycoplasma mastitis, since it could be highly contagious and unresponsive to any treatment (2). Culling animals is the most common way to control and prevent the spread of mycoplasma mastitis in the farm independently from the bacterial load and *Mycoplasma* species detected (3, 4). The documented transmission routes of *Mycoplasma* spp. are contaminated milking equipment and animal contact through respiratory secretions (5). Environmental sources of *Mycoplasma* spp. and their role in transmitting diseases are poorly known. To our knowledge, the few environmental sources where *Mycoplasma* spp. have been isolated on dairies are represented by cooling ponds, dry lots, and recycled bedding (6). Data from a study performed in Utah (7) showed that *Mycoplasma* spp. were found in 51% of the recycled sand bedding collected on dairy farms during outbreaks of clinical mastitis caused by *Mycoplasma* spp. The same study also showed that *Mycoplasma* spp. remained viable in recycled bedding sand for up to 8 months. Manure and other different bedding types represent the primary sources for houseflies’ nutrition and oviposition.

Houseflies represent a threat to dairy farms, resulting in significant losses attributable to the transmission of diseases, including mastitis (8, 9). To shed light on the potential role of flies in the diffusion of mycoplasmas, the present study investigated the genomic relatedness of a *M. arginini* isolated from a housefly captured in a dairy farm with eight *M. arginini* isolated from bovine milk samples and one isolated from bovine lung tissue. The *M. arginini* isolates of this study were obtained from different sampling periods ranging from months to years, even if specimens were collected from the same farm. In view of the uncertainty of *M. arginini* pathogenicity, we investigated the presence of 94 selected genes that putatively encode virulence factors (10). Since Mycoplasma bovis is the most prevalent, contagious, and clinically relevant *Mycoplasma* species in cattle, a set of pathogenicity genes was selected based among those already described for M. bovis, while another set was selected from the publicly available *M. arginini* genomic information (11).

## RESULTS

### Mycoplasma arginini isolation and identification.

In September 2019, we carried out a study on pathogens isolated from houseflies (Musca domestica) captured in different locations of a New York State dairy farm (12). The farm was selected based on a client list of the daily milk sample pick-up, 24h result program of Quality Milk Production Services (QMPS) and the history of cow udder pathogens isolated from milk, including S. aureus, *Mycoplasma* spp., *Prototheca* spp., and *Lactococcus* spp. Out of the 485 bacterial isolates obtained in that study, an isolate originating from the gut of a female housefly captured in the sick pen was identified as *M. arginini* by means of a multiplex-PCR assay associated with amplicon Sanger sequencing (13). Here, we present the detailed genomic analysis of this *M. arginini* isolate and evaluate its similarity with other isolates from bovine specimens.

### Genomic DNA sequencing, assembly, and annotation of the housefly gut *M. arginini* isolate.

**FIG 1:**
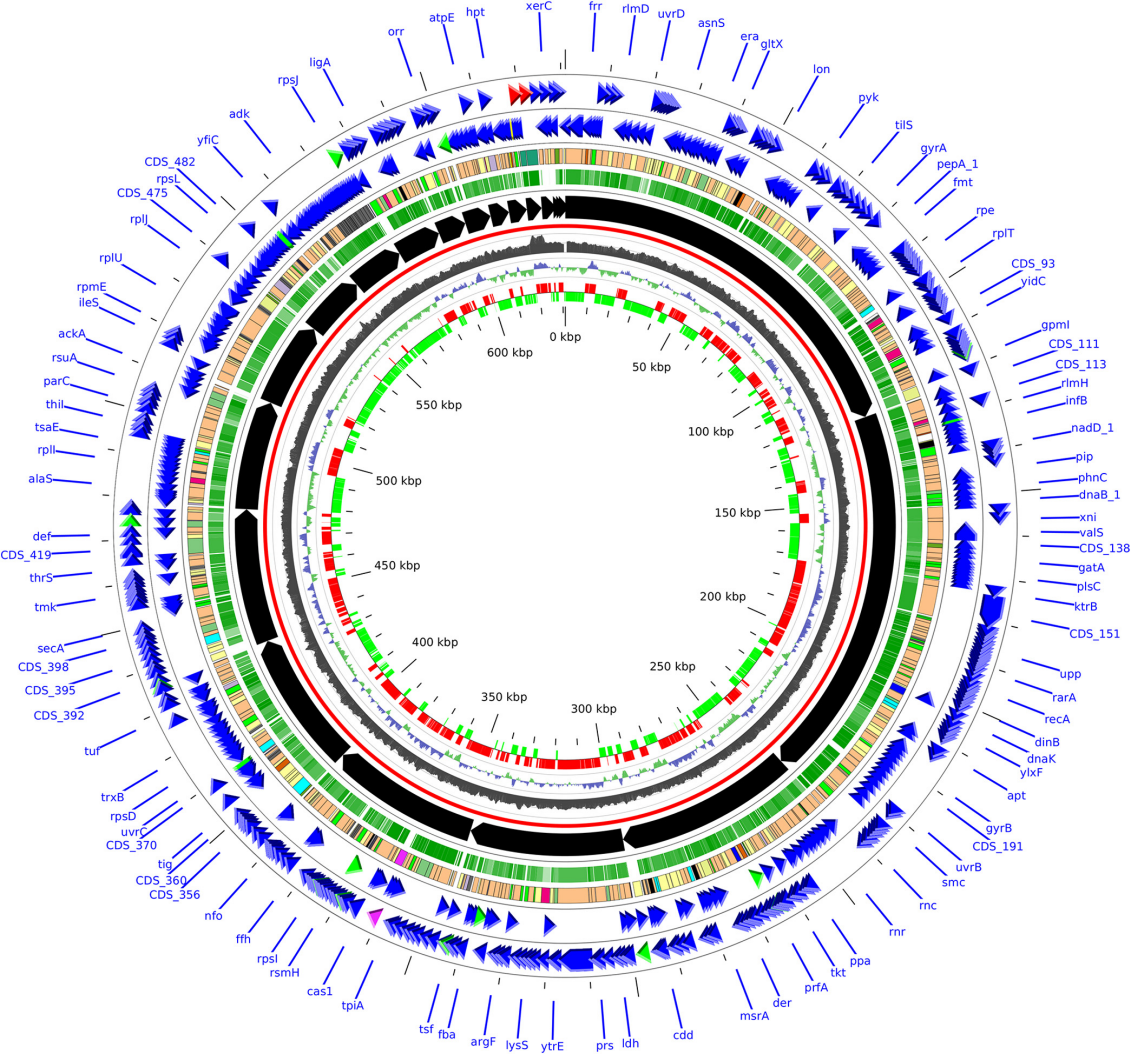
Circular plot of the draft genome of the *M. arginini* strain isolated from the fly gut. The 21 annotated scaffolds sum up to 631,023 bp. Each circle represents a feature of the genome: from the innermost circle, we represented: gene strand (red: positive, green: negative), the GC skew, the GC content, reference backbone (in red), scaffolds (ordered by decreasing length), the percentage of identity with *M. arginini* HAZ145 reference strain (opacity is proportional to % identity), CDS (colored by type; among them, light orange: enzymes, light yellow: hypothetical proteins), CDS on negative and positive strands (colors indicate: blue: proteins, red: rRNA, green: tRNA).

Genomic DNA sequencing and assembly of the housefly gut *M. arginini* isolate generated 21 scaffolds of length >500 nt, for a total of 631,023 bp; the maximum scaffold length was 122,725 bp, with 16 out of the 21 scaffolds above 5Kb and an *N*_50_ of 48,510 bp. GC content overall the scaffolds was 26.3%. Genome size and GC content were comparable to those of the reference genome HAZ145. The assembly included a total of 584 genes, including two rRNAs (16S and 23S), 32 tRNAs, 1 transfer-mRNA (tmRNA), 1 repeat region (CRISPR-associated with 32 repeat units), and 548 protein-coding genes. A hundred and eight CDS (19.7%) were annotated as “hypothetical proteins” of unknown function. Two hundred sixty-five out of the 548 protein-coding genes (48.4%) were classifiable as enzymes (e.g., nucleases, proteases, hydrolases), 14 as ATP-binding genes, 10 as lipoproteins, and 51 as ribosomal proteins. Among enzymes, the most represented were transferases (44 genes), tRNA ligases (21 genes), permeases (14 genes), nucleases (15 genes), peptidases (12 genes), and hydrolases (11 genes). Five hundred eighty-three features (99.8% of the total features) had a corresponding match on *M. arginini* reference strain HAZ145, with very high similarity (average identity of 99.1% ± 1.4% on a nucleotide basis) and covering nearly all the gene sequences (average coverage of 99.1% ± 7.9%) (Fig. 1).

Five hundred seventy-eight out of the 591 CDS (97.8%) of the HAZ145 reference genome found a match in the housefly gut isolate assembly; 10 out of the 13 missing genes were annotated as hypothetical proteins, 1 was a very short peptide (a 30 aa type II restriction endonuclease) and 1 more was reported as a pseudo-gene in HAZ145.

### Genomic DNA sequencing of *M. arginini* strains isolated from milk and lung tissue.

With the aim of understanding the relationships of the housefly gut isolate with other sources of *M. arginini*, we compared it to nine isolates of the same species from the same and other New York State farms and stored in our institutional repository. Specifically, we selected two milk isolates from the same farm, six milk isolates from other farms, and one bovine lung isolate. These were subjected to whole-genome sequencing. Isolates and sequencing data are reported in Table 1.

**TABLE 1:** Isolates included in this study and relative sequencing data

Isolate ID	Date	Source	Farm	Genome size^*a*^	N. contigs	Avg. length^*b*^	Largest contig length^*b*^	*N*_50_	Accession no.
20069D-01-02	2019-9	Fly gut	A	634,492	37	17,148	122,725	48,510	JAJPGJ010000000
20069D-02-01	2017-3	Milk	A	476,969	602	792	6,746	1,025	n.a.^*c*^
20069D-03-04	2017-4	Milk	A	632,854	29	21,823	122,725	48,509	JAPFAS000000000
20069D-02-03	2017-9	Milk	B	835,775	155	5,392	118,305	40,685	JAPFAV000000000
20069D-03-07	2017-11	Milk	B	778,237	244	3,190	118,309	32,471	JAPFAP000000000
20069D-03-02	2020-1	Milk	C	679,184	117	5,805	124,054	36,829	JAPFAU000000000
20069D-03-03	2020-1	Milk	C	745,228	264	2,823	124,054	35,571	JAPFAT000000000
20069D-03-05	2017-6	Milk	D	979,266	726	1,349	142,985	29,222	JAPFAR000000000
20069D-03-06	2018-2	Milk	E	799,647	300	2,665	260,840	39,661	JAPFAQ000000000
20069D-03-08	2018-9	Lung tissue	F	708,223	119	5,951	100,334	30,468	JAPFAO000000000

a Sum of the lengths of the individual scaffold/contigs of each assembly.

b Lengths in base pairs.

c See Data Availability statement.

As a result, eight out of the nine isolates (seven from milk and one from lung tissue) had an estimated genome size between 679,184 and 979,266 bp, all had similar average contig lengths (except 20069D-03-04), with an *N*_50_ comparable also to the housefly gut isolate; isolate 20069D-02-01 was an outlier, being the least sequenced one (genome size of 476,969 bp, a number of contigs three–times higher the average of the other strains, an average contig length of only 792 bp and a *N*_50_ considerably lower than the others).

### Phylogenetic tree generation based on 16S rRNA.

**FIG 2:**
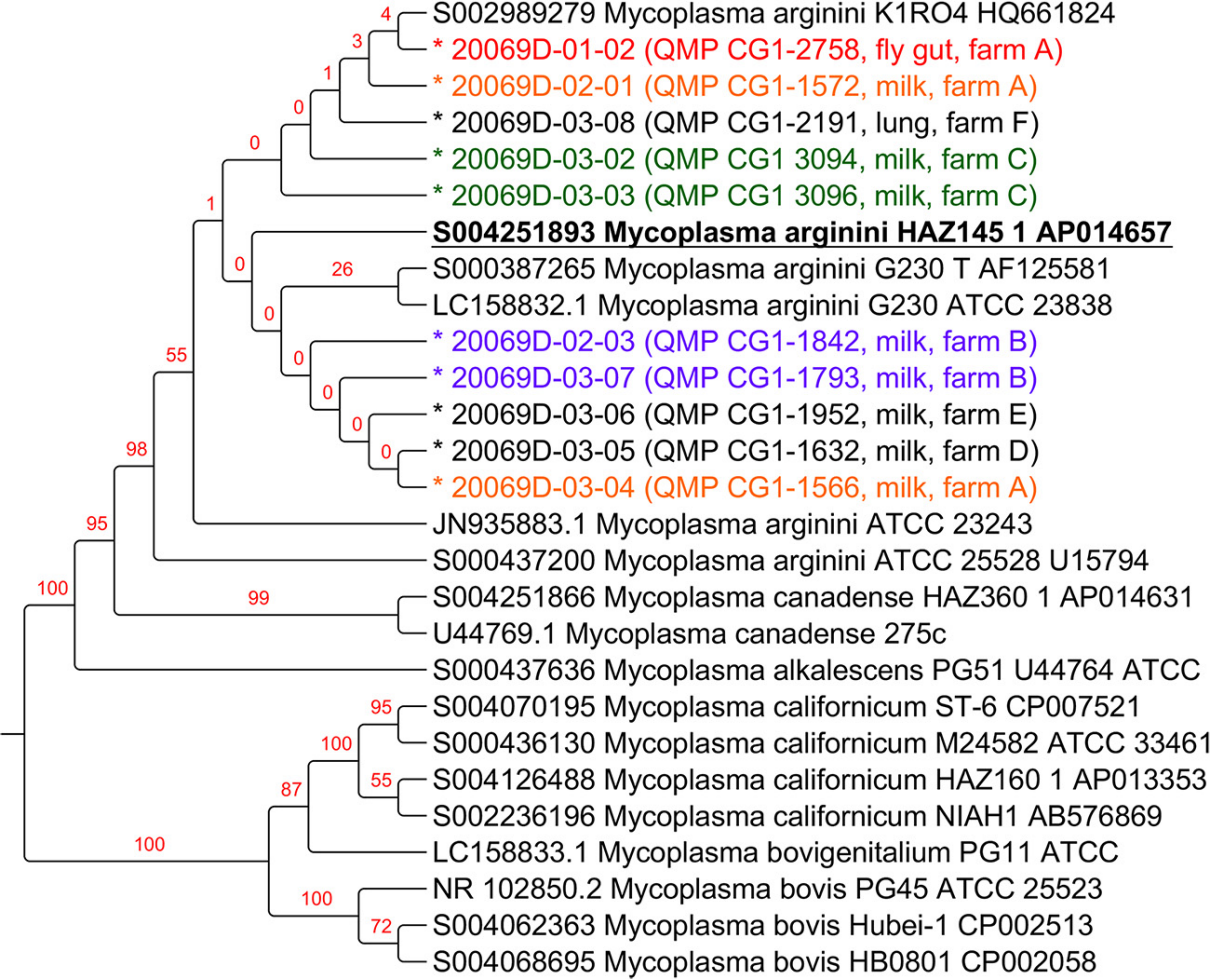
Maximum likelihood phylogenetic tree among 27 16S rRNA sequences from *M. arginini* and related species (*M. canadense*, *M. bovigenitalium*, M. bovis, *M. alkalescens*, *M. californicum*). Values from 100 bootstraps are indicated in red over the branches. Bootstrap values indicate how many times the same branch is observed when repeating the generation of a phylogenetic tree on a resampled set of data made by artificial sequences of randomly selected sites of the original sequences with replacement; this produces a different composition of the data with the same sequence length as the original data sets. For the newly sequenced isolates (indicated by a prepending “*”), the farm and source of isolation is indicated; colors in the plot help highlighting isolates from the same farms: red = fly gut; orange = milk from the same farm as the fly gut one (i.e., farm A); Green, purple = paired isolates from the other farms. The 16S rRNA from the HAZ145 reference sequence is highlighted in bold and underlined.

A phylogenetic tree was generated based on 16S rRNA sequences. Adding to the newly sequenced *M. arginini* isolates and the reference strain, the tree included 17 additional publicly available sequences from *M. arginini* and related species (*M. canadense*, *M. bovigenitalium*, M. bovis, *M. alkalescens*, *M. californicum*), for a total of 27 16S rRNA sequences. The results are illustrated in Fig. 2.

All the newly sequenced strains grouped in the *M. arginini* branch of the tree, which, at higher levels, also encompassed *M. canadense* and *M. alkalescens*, all grouped according to their species; on the other branch of the tree, again grouped according to species, were *M. californicum*, *M. bovigenitalium,* and M. bovis. Within the *M. arginini* branch, some strains derived from the same farm were close one to each other, although with low bootstrap values, such as 20069D-03-02 and 20069D-03-03, isolated from farm C, in green, 20069D-02-03 and 20069D-03-07, isolated from farm B, in purple. Interestingly, the housefly gut strain 20069D-01-02, isolated in farm A, in red, and the milk isolate 20069D-02-01 from the same farm A, in orange, were close in the phylogenetic tree. The other milk isolate coming from farm A, in orange, clustered in the same branch of the tree but farther from the housefly gut isolate.

By evaluating the identity of 16S rRNA genes, all *M. arginini* sequences were very similar to each other with identity >98.3%. *M. canadense* and *M. alkalescens*, which were placed in the same macro-branch of the 16S rRNA tree, were very similar to *M. arginini* (identity >97.2%). On the other hand, similarities of *M. arginini* with *M. californicum*, *M. bovigenitalium,* and M. bovis were lower (<87.5%). The similarity between M. bovis, *M. californicum,* and *M. bovigenitalium* was around 94%, with the latter more similar to *M. californicum* (identity >97.3%) than to M. bovis (Fig. S1).

### Phylogenetic classification refinement with conserved proteins in the newly sequenced strains.

**FIG 3:**
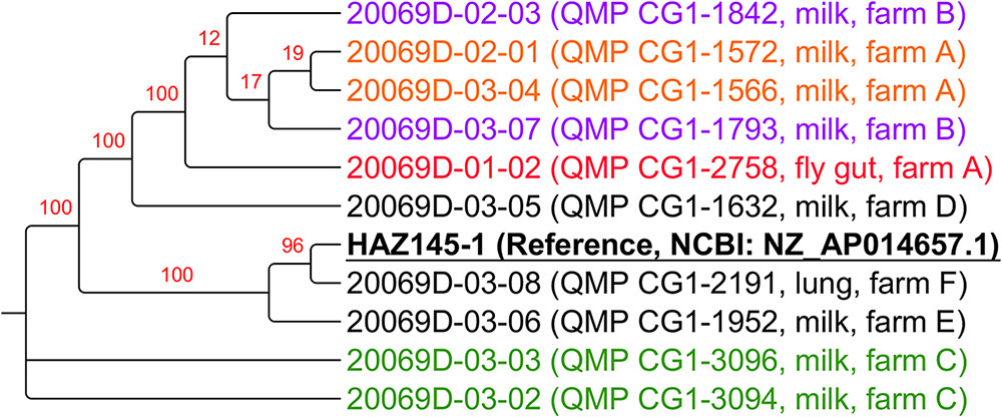
Maximum likelihood phylogenetic tree obtained creating an artificial sequence made up of 76 conserved genes among the 10 newly sequenced *M. arginini* strains and the HAZ145 reference genome. Values from 100 bootstraps are indicated in red over the branches. Bootstrap values indicate how many times the same branch is observed when repeating the generation of a phylogenetic tree on a resampled set of data made by artificial sequences of randomly selected sites of the original sequences with replacement; this produces a different composition of the data with the same sequence length as the original data sets. For the newly sequenced strains, the farm and source of isolation is indicated; colors in the plot help highlighting isolates from the same farms: red = fly gut strain; orange = milk-isolated strains from the same farm as the fly gut one (i.e., farm A); Green, purple = paired strains from the same farm. The HAZ145 reference sequence is highlighted in bold and underlined.

The phylogenetic classification of *M. arginini* strains was refined by employing a subset of 49 conserved proteins in the HAZ145-1 reference genome and in all the 10 newly sequenced strains, including the housefly gut isolate to generate a maximum likelihood phylogenetic tree. The results are reported in Fig. 3.

The phylogenetic tree of all 11 *M. arginini* strains based on the conserved protein sequences displayed four branches. In the first branch, the housefly gut isolate (20069D-01-02, red) clustered together with the two milk isolates from the same farm (A), which had been isolated from different cows 2 years earlier at different sampling times (20069D-02-01 and 20069D-03-04, orange). This branch also included the two milk isolates from farm B (20069D-02-03 and 20069D-03-07, purple). The milk isolates from farm C (20069D-03-02 and 20069D-03-03, green) clustered together on a separate branch of the tree. The milk isolate of *M. arginini* from farm E (20069D-03-06, black) was also distant from all the other newly sequenced isolates, clustering on the same branch with the reference strain HAZ145-1, isolated from bovine milk in Japan, and the bovine lung isolate from farm F (20069D-03-08, black). The milk isolates of farm D (20069D-03-05) fell in one branch equally distant from all the others.

**FIG 4:**
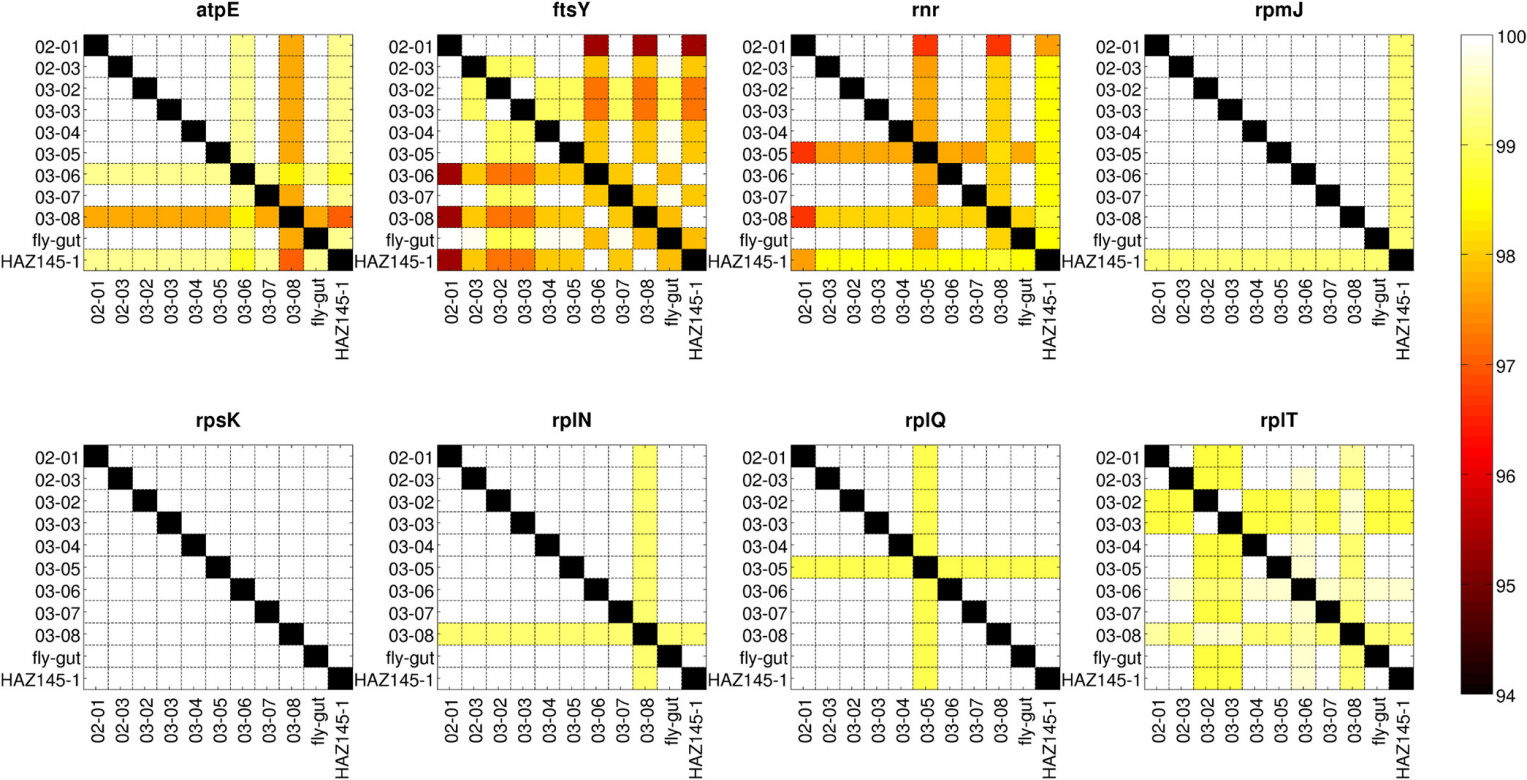
Representative heatmaps illustrating the percentage of identity (BLASTN value) between the 10 newly sequenced *M. arginini* strains and the HAZ145 reference genome. In the figure, 8 out of the 76 genes of the core genome are reported as representative of the different degree of identity among strains. The complete set of heatmaps can be found in Fig. S2 in the supplemental material −9.

The heatmaps representing the identity percentage for the single genes among all *M. arginini* isolates explain their clustering behavior in the phylogenetic tree. (Fig. 4) (Fig. S2 to 9). In fact, some genes (e.g., *rpsK*, *deoD*, *rpmF*, *upp*) displayed 100% identity among all isolates, whereas others (e.g., *ftsH*, *ftsY*, mutM, and *serS*) had lower identity values. In the tree, the reference genome clustered separately from the majority of the newly sequenced isolates (as for example, in *mnmA*, *mnmG*, *rpmJ* identity plots); on the other hand, the milk isolates from farms E and F (20069D-03-06 and 20069D-03-08, respectively) clustered together with the reference genome, in line with the *atpE*, *dnaJ,* and *dnaX* gene identity plots; *rplQ* and *rnr* also highlighted the distance of the farm D isolate (20069D-03-05) from all the others; on the other hand, the identity patterns of the two isolates from farm C (20069D-03-02 and 20069D-03-03) were extremely similar.

### Similarity analysis based on putative virulence genes.

With the purpose of investigating the virulence potential of the newly sequenced isolates, a total of 94 presumptive pathogenic genes were selected among those described in the literature (10) and aligned with all the *M. arginini* sequences. Of these, 35 (37%) had been previously reported for *M. arginini* and 59 (63%) for M. bovis.

**FIG 5:**
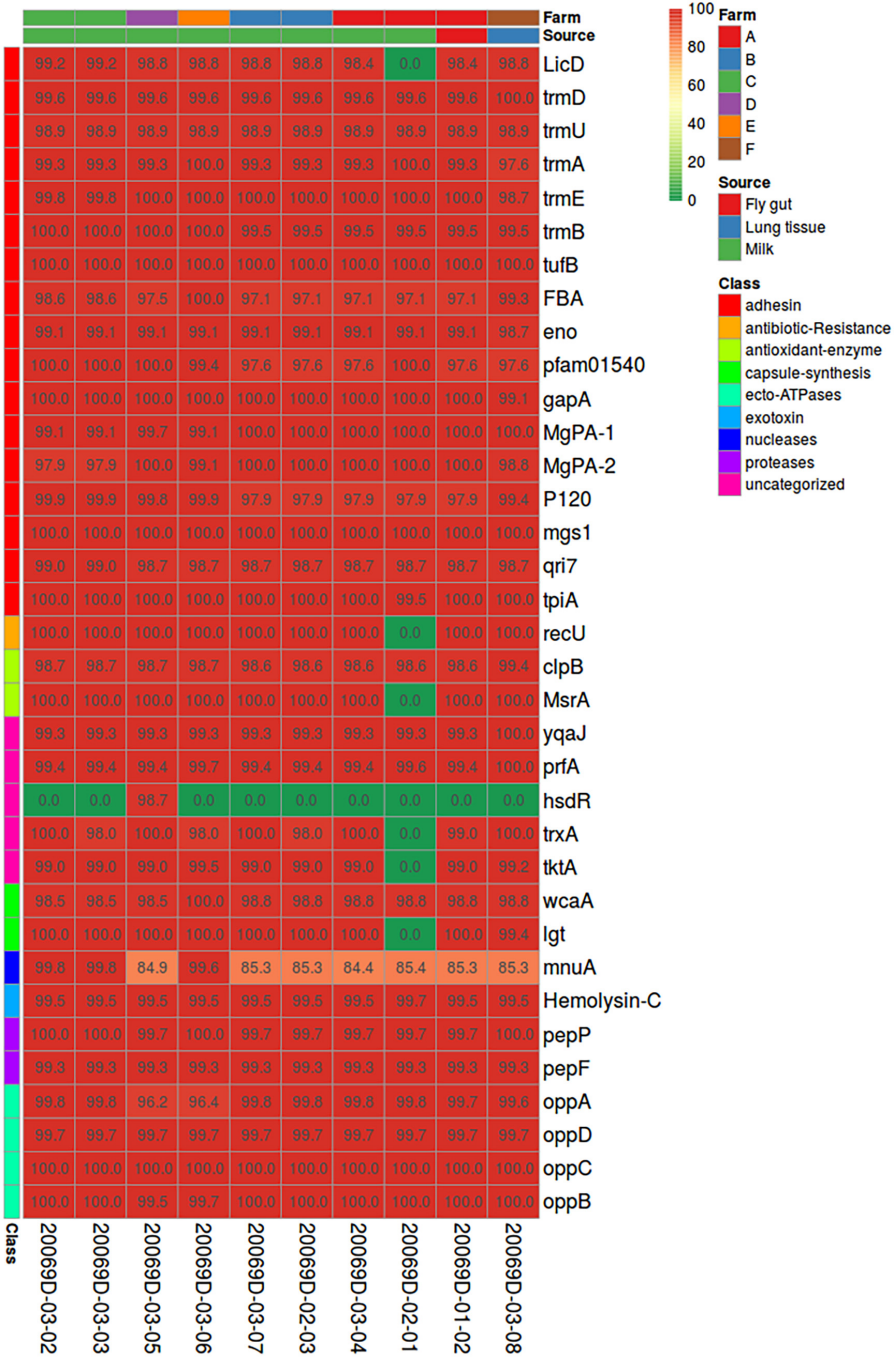
Heatmap representing the identity percentage (BLASTP value among translated nucleotide sequences) for a set of 35 pathogenicity-related genes found in the *M. arginini* reference strain HAZ145 over the 10 newly sequenced *M. arginini* isolates. Color is proportional to identity. Color bars over columns indicate the isolate origin and source; color bars to the left of each row represent the gene category.

All the *M. arginini* isolates sequenced in this study carried 34 out of the 35 pathogenicity genes present in the *M. arginini* reference strain (HAZ145), all with very high protein sequence identity percentages (>98%). The only exception was *hsdR* (type I restriction enzyme endonuclease), missing in all but one of the newly sequenced isolates: 20069D-03-05 (milk, farm D), which showed the gene in its entirety (identity: 98.7). The lack of some genes (i.e., LicD, recU, MsrA, trxA, tktA, lgt) in 20069D-02-01 isolate (milk, farm A) was most likely due to the incomplete sequencing of this strain (Fig. 5).

**FIG 6:**
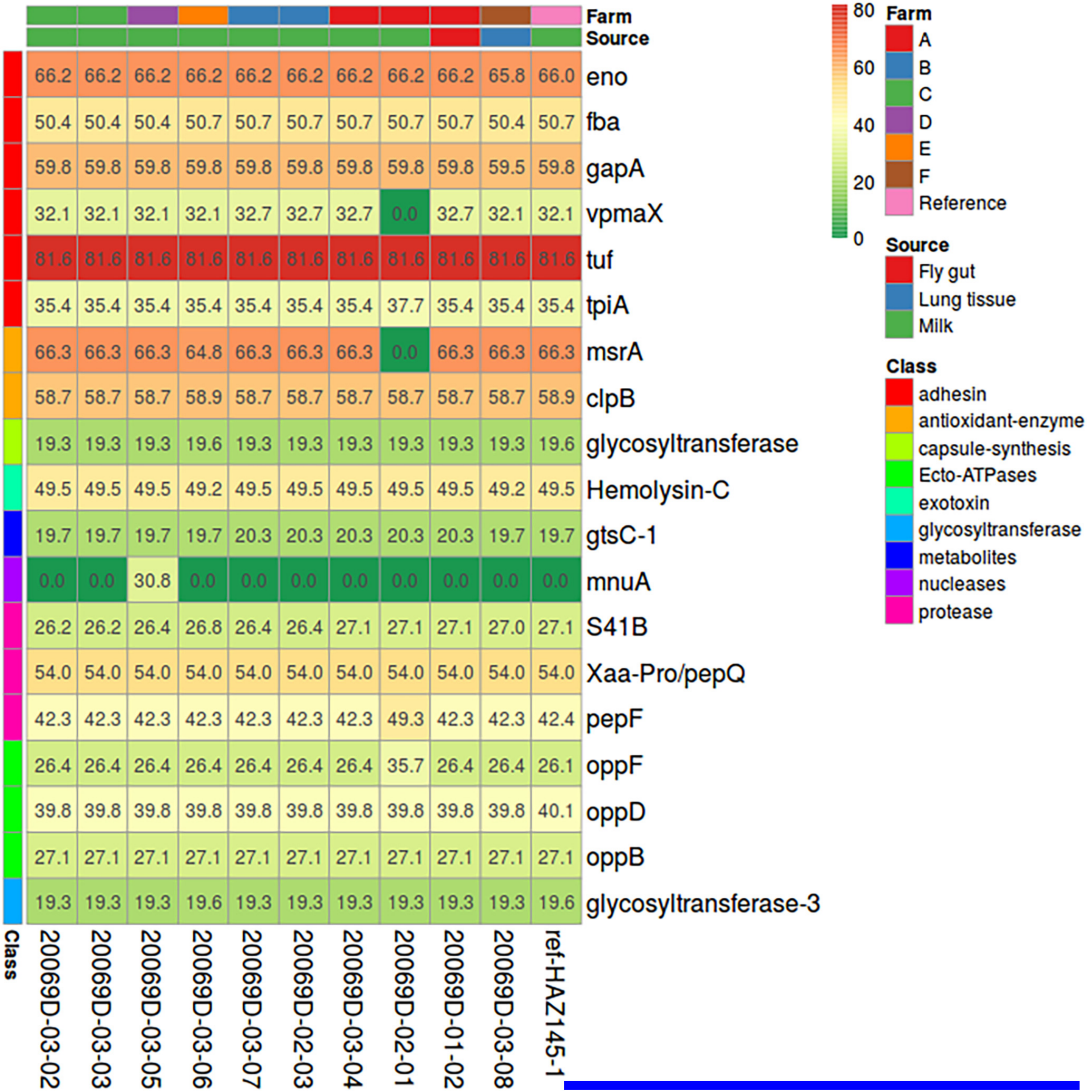
Heatmap representing the identity percentage (BLASTP value among translated nucleotide sequences) for a set of 59 pathogenicity-related genes found in the M. bovis strain PG45 (ATCC 25523) over the 10 newly sequenced *M. arginini* strains and the *M. arginini* reference strain HAZ145. Only 19 out of the 59 genes had a reliable match, whereas 13 others had a partial and 27 had no matches. Color is proportional to identity. Color bars over columns indicate the origin of the strain and the source the strain was extracted from; color bars to the left of each row represent the gene category.

Due to the scarce information regarding *M. arginini* pathogenicity and the limited number of isolates already characterized, M. bovis was also selected as a comparator. As a result, the presence of M. bovis derived pathogenicity genes was dramatically lower: only 19 out of the 59 selected genes had a match with those of the newly sequenced *M. arginini* isolates (Fig. 6). Identity levels were generally low, in the range 19% to 82%; the genes with the highest similarities were *tuf* (identity of 81.6%), *eno*, *gapA*, *clpB,* and *msrA* (identities in the range of 59%–67%). Notably *mnuA* was lacking in all isolates with the only exception for 20069D-03-05 (milk, farm D). When present, the identity of the newly sequenced isolates with M. bovis pathogenicity genes was the same as that of the reference genome HAZ145.

## DISCUSSION

To our knowledge, there are no previous reports of the association between bovine *Mycoplasma* spp. and any species of flies. On the other hand, *M. arginini* has been isolated from ruminant livestock’s respiratory tract and milk samples (11, 14). *M. arginini* is not one of the most frequent and pathogenic *Mycoplasma* species, but it can persist for a long time under different environmental conditions (15, 16). From other reports, no precise data suggest that *M. arginini* causes clinical mastitis, but it is often found in herds affected by mastitis associated with different etiologies. In a recent publication (4), *M. arginini* has been found to represent a 1.7% share of all mycoplasmas isolated in US dairy herds; it was isolated most frequently from bulk tank samples of herds where other *Mycoplasma* spp. were also found. It has been suggested that *M. arginini* may predispose animals to develop more severe signs of mastitis when other udder infections occur. Understanding the root of mycoplasma transmission is crucial for reducing dairy farm losses (4). To our knowledge, houseflies had never been considered possible carriers of *Mycoplasma* spp., contributing to their dissemination between animals or in the dairy environment. Despite the space-time interval linked to the dairy farms’ locations and to the multiple-year sampling, our results showed that the *M. arginini* isolated from the housefly was highly similar to the *M. arginini* isolated from milk samples.

The 16S rRNA-based phylogenetic tree of all Mycoplasma species sequences used for the present study identified two macro-branches. One of those located *M. arginini* isolates in the same macro-branch with *M. canadense* and *M. alkalescens*, similar to each other. On the other hand, *M. californicum*, *M. bovigenitalium* and M. bovis clustered in a different macro-branch distant from *M. arginini*. These results confirm what has been previously described by other authors concerning the phylogenesis of *Mycoplasma* spp. (17, 18). The same tree also placed our newly sequenced *M. arginini* isolates in the same branch as other published *M. arginini* sequences. However, our *M. arginini* isolates were located closer to each other, probably due to their shared country of origin (i.e., USA), whereas all other *M. arginini* 16S sequences in public databases were from other countries (e.g., Japan, South Africa). Within our isolates, there was also a tendency of the strains coming from the same farm to cluster together. Of particular interest was the very close clustering of the fly gut isolate with milk isolates from the same farm, even if those originated from samples collected 2 years earlier (farm A). In order to better refine the phylogenetic classification of the *M. arginini* isolates, we used a subset of 76 conserved proteins between the reference genome HAZ145-1 (12) and the isolates here sequenced. In this case, the housefly gut *M. arginini* isolate clustered together with four milk isolates originating from two of the six farms under evaluation. Most interestingly, two of them were from the same farm (farm A).

Such high similarity was especially notable since milk isolates had been obtained approximately 2 years earlier, and all the isolates from the other farms had been collected closer in time to the fly gut isolate. These findings suggest a relationship of the fly gut *M. arginini* isolate with the milk isolates from the same farm. The other two milk isolates clustering with the housefly gut isolate were from farm B. Milk isolates from farms C, D, and E, on the other hand, clustered in a distant branch from the fly gut isolate, and the two milk isolates from farm C clustered together on a separate branch of the tree, indicating a closer genetic makeup. The reference strain HAZ145-1, despite being described as isolated from milk, clustered with the lung isolate newly sequenced by our laboratory. The *M. arginini* isolated from the milk of farm E was distant from all other milk isolates but was closer to the reference strain and isolate F from the lung sample. Unfortunately, we have no data concerning exchange of cows or other factors behind the similarity between the two farms.

The 94 genes selected to characterize the *M. arginini* isolates of this study encoded 11 putative classes of virulence factors already described for *Mycoplasma* or other organisms. Instead, one group of genes was not associated with a particular class of bacterial virulence factors, despite being more related to viral or defense mechanisms (19–22) and was reported as an uncategorized class. We here evaluated the presence of genes encoding virulence factors that included (i) adhesins,(ii) antioxidant enzymes, (iii) capsule synthesis, (iv) ecto-ATPases, (v) exotoxins, (vi) nucleases, (vii) proteases, (viii) lipolytic enzymes, (ix) metabolites, (x) glycosyltransferases, and (xi) antibiotic resistance.

In the newly sequenced *M. arginini* isolates, we found 58% (54 out of 94) of all the putative virulence genes evaluated. The majority of these (*n* = 35) matched with the sequences already annotated for *M. arginini* HAZ145. On the other hand, only 19 genes matched with M. bovis sequences, and most of them showed a poor similarity.

The *M. arginini* isolated from fly gut was similar to the other milk- and lung-derived isolates in terms of presence and identity percentage to virulence factors from both *M. arginini* reference strain HAZ145 and M. bovis. Adhesion is the first and necessary step for both colonization and infection of the host cell (10). Thirty-seven of the 94 (39%) virulence genes considered in this study were classified as adhesins. In the genome sequences of our *M. arginini* isolates, we identified all the 17 putative adhesins contained in the *M. arginini* HAZ145 genome; these genes included *LicD*, *trmD*, *trmU*, *trmA*, *trmE*, *trmB*, *tufB*, FBA, *eno*, *pfam01540*, *gapA*, MgPA-1, MgPA-2, P120, *mgs1*, *qri7*, *tpiA*. A good share of these putative adhesins are metabolic enzymes reported as involved in pathogenicity with moonlighting mechanisms (23). Among these, *LicD* is a family of proteins involved in phosphorylcholine metabolism. *Trm* genes are methyltransferase groups with a possible cytoadherence function in *Mycoplasma* spp. (24). FBA (fructose-1,6-bisphosphate aldolase) and *eno* (enolase) are glycolytic enzymes with a potential role in adherence, invasion, and persistent infections as previously described for M. hyopneumoniae, M. bovis (25) and M. pneumoniae (26). gapA (Glyceraldehyde-3-phosphate) is another metabolic enzyme with a possible role as cytadhesin in M. gallisepticum (27); as mgs1, it has been described as erythrocyte-binding adhesin in *M. suis* (10, 28)*. tpiA* (Triosephosphate isomerase) is a metabolic enzyme that serves as a critical interlink between glycolysis and glycerol metabolism and phospholipid synthesis. Some studies suggest that the surface-exposed TPIs also play a role in the pathogen–host interaction (29–31). It has been suggested by *in vitro* experiments that M. gallisepticum uses *tpiA* to cytoadhere the DF-1 cells (32, 33). Among the genes encoding adhesins within the M. bovis reference genome, the only one that showed high identity values (81.6%) for the newly characterized *M. arginini* isolates was *tuf* (Elongation factor TU, EF-Tu). EF-tu is one of the most abundant proteins in bacterial cells with a crucial role in protein translation (25, 34). However, EF-tu has also been reported with a cell surface localization as an important adhesin in many pathogens; it also appears to be the most immunoreactive protein and may be related to M. bovis biofilm formation (35). The biofilm growth provides M. bovis several advantages, including remarkable resistance to antimicrobials and persistence in the environment and host, which may result in the chronicity of a disease and considerable difficulty of treatment (36). EF-Tu is also a well-characterized extracellular matrix (ECM) binding adhesins for M. hyopneumoniae. The binding between EF-Tu with fibronectin contributes to the adhesion of M. hyopneumoniae in swine tracheal epithelial cells (STEC) (25). Being involved in crucial metabolic cell functions, the possible role of the above discussed housekeeping proteins in *M. arginini* pathogenicity through moonlighting functions will need to be investigated with specific, dedicated studies. Other proteins, however, are known for their primary role as adhesins. Based on the *M. arginini* HAZ145 genome annotation, pfam01540 gene codes for an adhesin lipoprotein (11). MgPA (putative MgPA-like protein) is a major adhesin mediating the attachment of the M. genitalium to the ciliated epithelium (10), resulting from a horizontal transfer from unknown origins. Both homologous MgPA-1 and MgPA-2 were found at high identity values to HAZ145 reference in all the new *M. arginini* isolates. *P120* is a surface membrane protein described as a putative virulence factor in *M. hominis* (37)*. P120* undergoes genetic variability that could account for the ability of the microorganisms to circumvent the host immune system.

Proteases contribute to bacterial virulence for their potential to destroy functional and structural proteins of the host defense barriers (38). A total of three genes (*pepP xaa-pro*, *pepF,* and S41B) coding for proteases have been included in our analysis; all of them have been identified in the genome sequences of the new *M. arginini* strains characterized in this study. Other genes coding for proteins with protease functions were Xaa-Pro aminopeptidase and qri7_ Metal-dependent proteases with possible chaperone activity (39).

Four genes coding for uncategorized virulence factors (i.e., *yqaJ*, *prfA*, *trxA,* and *tktA*) were found in the characterized *M. arginini* isolates, including the one isolated from the fly gut. A fifth uncategorized virulence factor (*hsdR*) was missing in the fly gut *M. arginini* genome and in eight out of the other nine isolates. Another gene, *recU*, found in all but one the *M. arginini* genomes analyzed in this study, is reported to be involved in antimicrobial resistance of S. aureus (40) and Bacillus subtilis (41), and has a main role in DNA repair and homologous recombination, which is involved in the transfer of DNA within and between species and can lead to acquisition of new traits that can increase virulence or antibiotic resistance. Further studies will be needed to understand the potential role of these genes in *M. arginini* virulence.

In general, mycoplasmas lack the biosynthetic pathways for cholesterol, fatty acids, some amino acids, purines, and pyrimidines and, therefore, acquire these through membrane transport proteins (42). *Mycoplasma* genomes possess a diversity of ABC transporters predicted to be involved in the uptake of these inorganic and organic substrates (43). In the fly gut-derived *M. arginini* and all the other genomes analyzed in this study, the oligopeptide ABC transporter system was synthesized in the *opp* operon cluster genes (*oppA*, *oppB*, *oppC*, *oppD* genes). These uptake systems are mainly involved in cell nutrition; however, some might be associated with virulence (44, 45).

Notably, the *in-silico* virulence gene profiling highlighted that the housefly *M. arginini* isolate was genetically comparable to the other *M. arginini* isolated from bovine milk and lung samples. This is critical to define houseflies as reservoirs and carriers of *M. arginini*. We should consider that the contagiousness level of *M. arginini* is not known, and there is not much information available from the literature. Still, most dairy herds have zero tolerance for *Mycoplasma* spp., and the animals resulting positive are culled independently from which *Mycoplasma* species are isolated from milk samples. The gold standard detection methods rely on bacterial cultures showing colonies with mycoplasma morphology, and most of the time, dairy management decisions in the case of positive cows are based on culture results without going for any further speciation. At this point, it is important to reconsider the environment as a possible source of *Mycoplasma* spp. in dairies and in particularly houseflies as their carriers.

More *in vitro* or *in vivo* studies will be needed to confirm our findings based on *in-silico* analysis and to establish the mechanism of *Mycoplasma* transmission between houseflies and dairy cows. Since the new *M. arginini* strain characterized here has been isolated from the gut of a housefly and not from its exoskeleton, transmission by mechanical translocation can be excluded for the conditions of the present study. Here, transmission routes are most likely represented by flies regurgitation (bio-enhanced transmission) and or defecation (46). It is essential to consider that microbial contamination from the dairy environment also includes residues of milk, from either healthy or diseased cows, that could persist in the environment due to poor sanitation and hygiene dairy practices. Milk residue on utensils and milking equipment represent feeding elements for flies (47). Once fed and when flies find another food source or place to lay eggs, the crop fluids are either externally released by regurgitation or ingested internally to the gut to be then re-emitted into the environment by defecation. These findings reinforce the importance of mycoplasma diagnostics in cattle by performing culture on samples collected aseptically to ensure that what is detected is a natural agent of infection, not a contaminant.

### Conclusion.

The data presented here support the hypothesis that houseflies could be potential carriers of *Mycoplasma* spp. and need to be considered within the possible roots of environmental transmission. Further studies with a higher population of flies are required to confirm our hypothesis and evaluate if other *Mycoplasma* spp. can be isolated from houseflies. Concerning the potential influence of *M. arginini* on mammary gland health, the housefly isolate possessed all the pathogenicity genes identified in the reference strain and shared a low number of virulence genes with M. bovis.

## MATERIALS AND METHODS

### Housefly sampling and homogenization.

The housefly that carried *M. arginini* isolate, the focal point of this study, was one of the specimens collected from our research survey conducted in 2019 on a commercial dairy farm in New York State. Methods for houseflies sampling and homogenization are not described in the manuscript but in the referenced paper (12).

### Milk samples.

*M. arginini* isolates found in bovine milk originated from eight samples submitted to the Quality Milk Production Center (QMPS) at Cornell University for *Mycoplasma* testing. Milk samples were received from four different dairy farms in New York State. Milk samples were submitted to the QMPS labs over 3 years (2017 to 2020). Most milk isolates, 7 of 8 (87.5%), were collected from composite samples, and 1 of 8 (12.5%) was obtained from an individual quarter sample. Composite samples were tested for herd surveys or screening of fresh cows, while the quarter sample was collected from a case of clinical mastitis.

### Lung tissue sample.

The *M. arginini* isolate found in bovine lung tissue was collected from a chronic pneumonia case submitted for *Mycoplasma* testing to the Bacteriology Laboratory of the Animal Health Diagnostic Center (AHDC) at Cornell University.

### Mycoplasma testing.

*Enrichment:* The *Mycoplasma* enrichment step was performed only for the fly sample after being processed by the homogenization step described above. A volume of 0.5 mL from the fly’s suspension were transferred in 1 mL of Mycoplasma Broth (Hardy Diagnostics, Santa Maria, CA) and incubated for 72 h at 35°C to 38°C with 5 to 10% CO_2_. After the incubation, the sample was submitted for mycoplasma culture. As enrichment step, the sample was inoculated using cotton swabs on Mycoplasma Hayflick Agar (Northeast Laboratory) and incubated for 7 days at 37°C.

*Culture:* All samples, independently from the type of specimen, were mixed by inversion and then streaked by sterile cotton swab on Mycoplasma Hayflick Agar (Northeast Laboratory) and incubated for 7 days at 35 to 38°C with 5 to 10% CO_2_. *Mycoplasma* growth was detected by visual inspection of culture plates under an illuminated stage stereomicroscope. *Mycoplasma* colonies were confirmed by a PCR specific for *Mycoplasma* and *Acholeplasma* followed by Sanger sequencing for species identification (13).

### Library preparation.

Library preparation and sequencing were performed by Admera Health (South Plainfield, NJ, USA). Isolated genomic DNA was quantified with Qubit 2.0 DNA HS Assay (ThermoFisher, Waltham, MA, USA) and DNA quality was evaluated by Genomic DNA ScreenTape analysis (Agilent Technologies, Santa Clara, CA, USA). Library preparation was performed using Nextera XT library kit (Illumina, San Diego, CA, USA) following the manufacturer’s recommendations while barcoding was performed using Illumina 8-nt dual-indices. The final library was evaluated by Qubit 2.0 DNA HS Assay and the quality of the library generated was examined by TapeStation HSD1000 ScreenTape (Agilent Technologies). Final library quantity was measured by KAPA SYBR FAST qPCR with QuantStudio 5 System (Applied Biosystems, Waltham, MA, USA). Equimolar pooling of libraries was performed based on qPCR QC values before sequencing on an Illumina HiSeq with a read length configuration of 2 × 150 paired end reads, obtaining 1 M PE reads per sample.

### Genome assembly and annotation.

For the gut fly-isolated strain, as well as for milk- (*n* = 8) and lung-tissue-isolated (*n* = 1) strains, assembly was conducted by Admera Health using SPAdes (v. 3.14.0) with parameter “-k 127.” SPAdes was also used for generating genomic scaffolds on gut fly strain by using paired-end reads in the sequencing to concatenate contigs (48). The initial functional annotation on all sequenced strains was initially performed by prokka (v. 1.14.6) (49), and further refined by using GView Server utility (https://server.gview.ca/) (50) in PanGenome mode, employing all newly sequenced genomes and an available reference sequence (i.e., Mycoplasma arginini, strain HAZ145_1, https://www.ncbi.nlm.nih.gov/nuccore/NZ_AP014657.1) as the template (11). Finally, a second optimization was performed by performing a protein BLAST+ (v. 2.11.0) (51) search against “nr” database and limiting matches only to *M. arginini*-derived proteins (additional parameters: -word_size = 6. -max_target_seqs = 5, -evalue = 10e-3). Genome features were plotted by GView (50).

### Phylogenetic tree building.

Phylogenetic analysis of the gut fly-isolated strain compared to other *Mycoplasma* species was inferred using the 16S rRNA genomic sequence as a marker. A total of 27 sequences were used for tree building: 16 *M. arginini* (including 10 from our assemblies and six from publicly available resources), 2 *M. canadense*, 1 *M. alkalescens*, 4 *M. californicum*, 1 *M. bovigenitalium*, and 3 M. bovis. (Table 2).

**TABLE 2:** List of 16S rRNA sequences used for building the phylogenetic tree

Mycoplasma species	Strain	Accession N.	Source
*M. arginini*	20069D-01-02^*a*^	JAJPGJ000000000 ^*b*^	Housefly gut
	20069D-02-01^*a*^	ON890822	Bovine Composite Milk
	20069D-02-03^*a*^	ON890823	Bovine Mastitis Milk
	20069D-03-02^*a*^	ON890824	Bovine Composite Milk
	20069D-03-03^*a*^	ON890825	Bovine Composite Milk
	20069D-03-04^*a*^	ON890826	Bovine Composite Milk
	20069D-03-05^*a*^	ON890827	Bovine Composite Milk
	20069D-03-06^*a*^	ON890828	Bovine Composite Milk
	20069D-03-07^*a*^	ON890829	Bovine Composite Milk
	20069D-03-08^*a*^	ON890830	Bovine Lung
	G230 / ATCC 23838	LC158832.1	Mouse brain with sheep scrapies
	G230(T)	AF125581.1	—
	HAZ145	AP014657.1	Bovine Mastitis Milk
	K1RO4	HQ661824	Vaginal swab from Dorper sheep
	ATCC 23243	JN935883.1	Sheep Lung with pneumonia
	ATCC 25528	U15794	Lion Lung
*M. canadense*	275c	U44769	Bos taurus
	HAZ360	AP014631	Bovine Mastitis Milk
*M. alkalescens*	PG51	U44764	Bovine Nasal cavity
*M. californicum*	ATCC 33461	M24582	Bovine Mastitis Milk
	NIAH1	AB576869	Bovine Mastitis Milk
	ATCC 33461T	CP007521	Bovine Mastitis Milk
	HAZ160_1	AP013353	Bovine Mastitis Milk
*M. bovigenitalium*	PG11 / ATCC 19852	LC158833	Bovine Genital Tract
M. bovis	ATCC 25523	NR_102850	Bovine Mastitis Milk
	HB0801	CP002058	Bovine Lung
	Hubei-1	CP002513.1	Bovine Lung

a Indicates the *M. arginini* strains sequenced in the present paper; —: not available.

b The sequence of 16S rRNA is contained in sequence CONTIG_17, bases 14–1531, feature id: KMZ28_00575. For each sequence, the species ID, the strain, the GenBank accession number and the source are indicated.

Identity among the 16S rRNA sequences was assessed by BLAST+. Phylogenetic reconstruction based on 76 genes conserved on all the 10 strains and the reference was performed using Phylogeny.fr utility (https://www.phylogeny.fr/) (52), using clustalO for multiple alignments, Gblocks for alignment curation, and PhyML for tree building, with 100 bootstraps. TreeGraph (v. 2.15.0-887 beta) was used to draw the final tree (53).

### Check of pathogenic genes.

A total of 94 presumptive pathogenic genes were selected among those described in the literature (10); 35 were previously reported for *M. arginini* and 59 for M. bovis. The nucleotide sequences were retrieved from NCBI GenBank and translated into protein using utility “transeq” from EMBOSS package (54). Protein sequences of the newly sequenced *M. arginini* strains were those predicted by prokka. BLAST+ (-task= blastp, -evalue 10e-3) and were employed to find best matches between references and queries, selecting only those spanning at least 75% of the reference or query (whatever the shorter of the two). The percentage of identity was plotted in a heatmap using ClustVis (https://biit.cs.ut.ee/clustvis/) (55).

### Data availability.

All raw data described in the paper are linked in NCBI BioProject database (accession number: PRJNA733869). Raw sequencing reads of the isolates are available within NCBI Short-Reads Archive (SRA) under accession number SRR17253962; 16S rRNA sequences are available in GenBank, under accession numbers: ON890822-ON890830. The Whole Genome Shotgun project of the fly gut-isolated *M. arginini* (20069D-01-02) has been deposited at DDBJ/ENA/GenBank under the accession JAJPGJ000000000. The version described in this paper is version JAJPGJ010000000. The Whole Genome Shotgun projects for the milk- and lung-derived *M. arginini* isolated have been deposited under accession JAPFAO000000000, JAPFAP000000000, JAPFAQ000000000, JAPFAR000000000, JAPFAS000000000, JAPFAT000000000, JAPFAU000000000, and JAPFAV000000000. Due to sequencing coverage issue, WGS for isolate 20069D-02-01 is missing; the sequences of the 76 genes used for the core genome analysis for this isolate are available in GenBank under accession numbers OQ078009-OQ078084. See also Tables 1 and 2 for more details.
