# Supplementary material for: Genomic Characterization of Mycoplasma arginini Isolated from a Housefly on a Dairy Farm and Comparison with Isolates from Bovine Milk and Lung Tissue
Source: Microbiol Spectr. 2023 May 18;11(3):e03010-22. doi: 10.1128/spectrum.03010-22 (PMC10269790; doi:10.1128/spectrum.03010-22)

SUPPLEMENTARY MATERIAL

Genomic characterization of *Mycoplasma arginini* isolated from a housefly in a dairy farm and comparison with isolates from bovine milk and lung tissue

G. Gioia,[1a] M. Severgnini,[2a] P. Cremonesi,[3a] B. Castiglioni,[3] J. Freeman,[4] A. Sipka,[1] C. Santisteban,[1] M. Wieland,[1] V. Alanis Gallardo,[1,5] J.G. Scott,[4] P. Moroni, [1,6b] M.F. Addis[6]

[1]Quality Milk Production Services, Animal Health Diagnostic Center, Cornell University, Ithaca, NY 14850, United States of America

[2] Institute of Biomedical Technologies, National Research Council, via f.lli Cervi, 93, 20054, Segrate, Milano, Italy

[3] Institute of Agricultural Biology and Biotechnology, National Research Council, via Einstein, 26900, Lodi, Italy.

[4] Department of Entomology, College of Agriculture and Life Sciences, Cornell University, Ithaca, NY, 14853

[5] Departamento de Medicina Preventiva y Salud Pública, Facultad de Medicina Veterinaria y Zootecnia, Universidad Nacional Autónoma de México, Ciudad Universitaria, CDMX 04510, México

[6] Università degli Studi di Milano, Dipartimento di Medicina Veterinaria e Scienze Animali, Via dell'Università, 6, 26900, Lodi, Italy

[a] These authors contributed equally

[b] Corresponding author: Paolo Moroni, pm389@cornell.ed

SUPPLEMENTARY FIGURES **3**

SUPPLEMENTARY FIGURE 1 3
SUPPLEMENTARY FIGURE 2 4
SUPPLEMENTARY FIGURE 3 5
SUPPLEMENTARY FIGURE 4 6
SUPPLEMENTARY FIGURE 5 7
SUPPLEMENTARY FIGURE 6 8
SUPPLEMENTARY FIGURE 7 9
SUPPLEMENTARY FIGURE 8 10
SUPPLEMENTARY FIGURE 9 11

Supplementary Figures

Supplementary Figure 1

Diagram depicting the pairwise percentage of identity (Blastn value) among the 27 16S rRNA sequences analyzed. Circle size is proportional to the identity, whereas the color graphically represents the identity: 100.0%, 95.0%-99.9%, 90.0%-94.9%, 85.0%-89.9%, ≤84.9%).

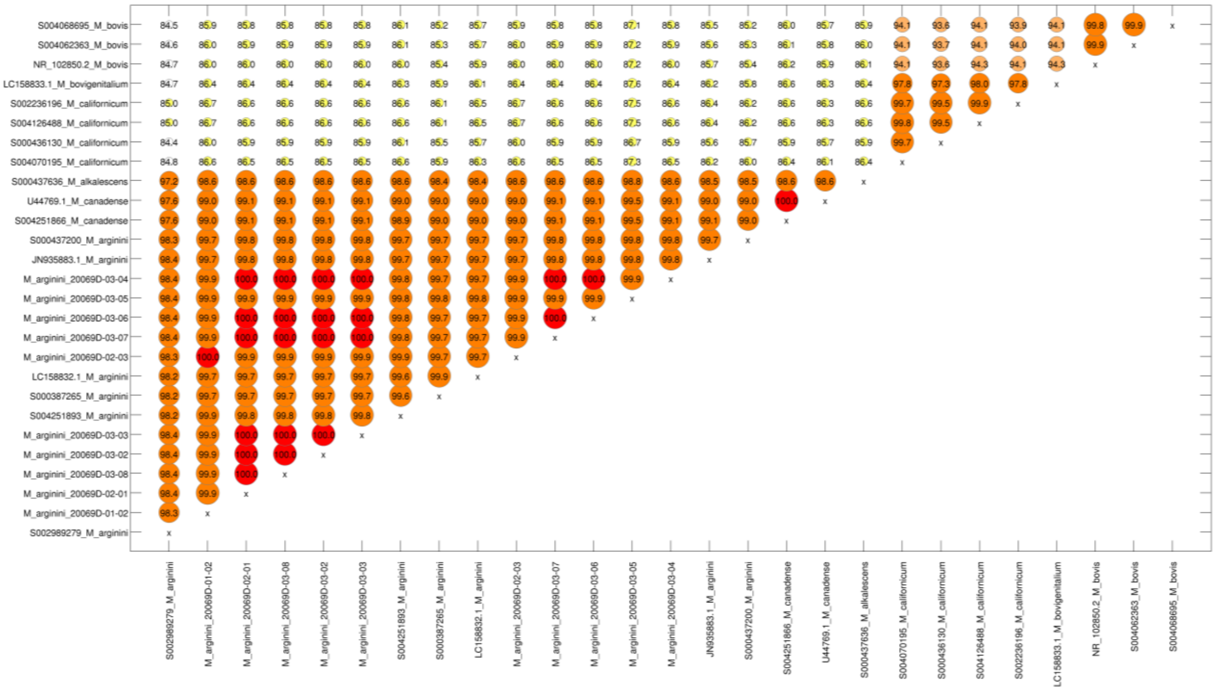

Supplementary Figure 2

Heatmaps illustrating the percentage of identity (Blastn value) between the 10 newly sequenced *M. arginini* strains and the HAZ145 reference genome. In the figure, genes arcA, argF, aspS, atpD, atpE, atpG, cmk, def, deoD, dnaB are represented. A representative set of genes can be found in Figure 4 of the main text

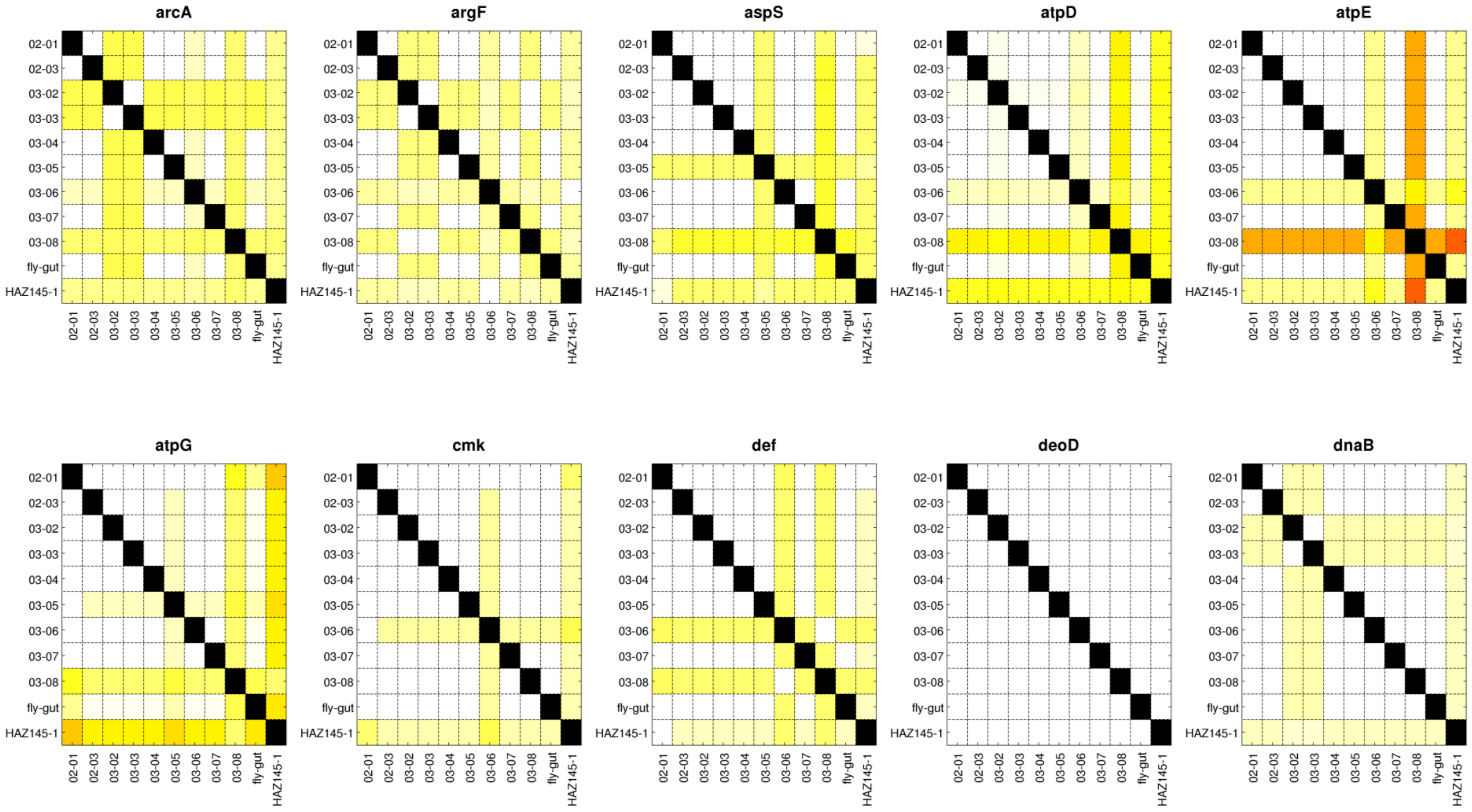

Supplementary Figure 3

Heatmaps illustrating the percentage of identity (Blastn value) between the 10 newly sequenced M. arginini strains and the HAZ145 reference genome. In the figure, genes dnaJ, dnaN, dnaX, era, ftsH, ftsY, gap, greA, gyrB, infA are represented. A representative set of genes can be found in Figure 4 of the main text

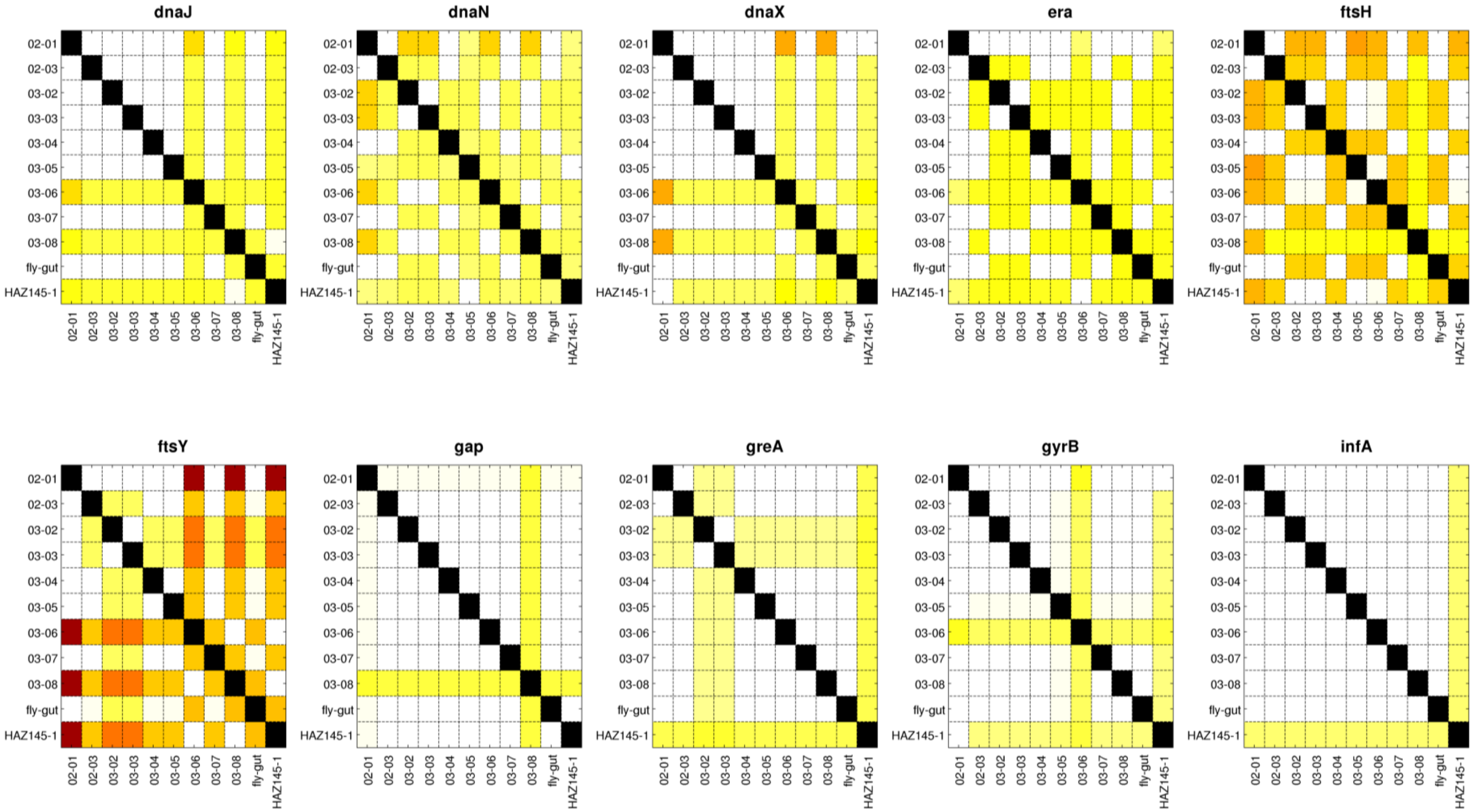

Supplementary Figure 4

Heatmaps illustrating the percentage of identity (Blastn value) between the 10 newly sequenced M. arginini strains and the HAZ145 reference genome. In the figure, genes lepA, lon, lysS, map, mnmA, mnmG, mutM, nadE, pepF, prfA are represented. A representative set of genes can be found in Figure 4 of the main text

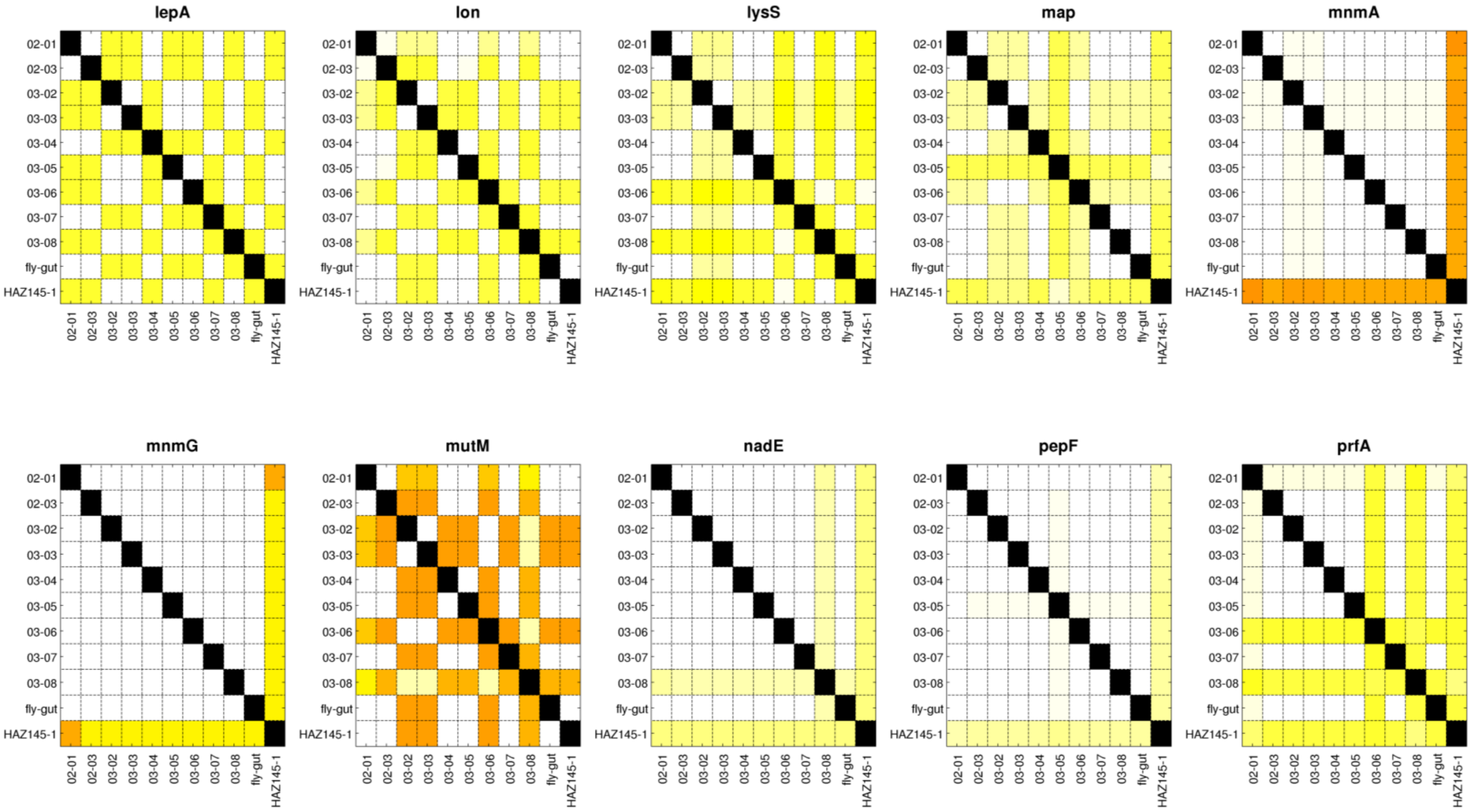

Supplementary Figure 5

Heatmaps illustrating the percentage of identity (Blastn value) between the 10 newly sequenced M. arginini strains and the HAZ145 reference genome. In the figure, genes pyk, recA, rnpA, rnr, rplB, rplC, rplD, rplE, rplF, rplK are represented. A representative set of genes can be found in Figure 4 of the main text

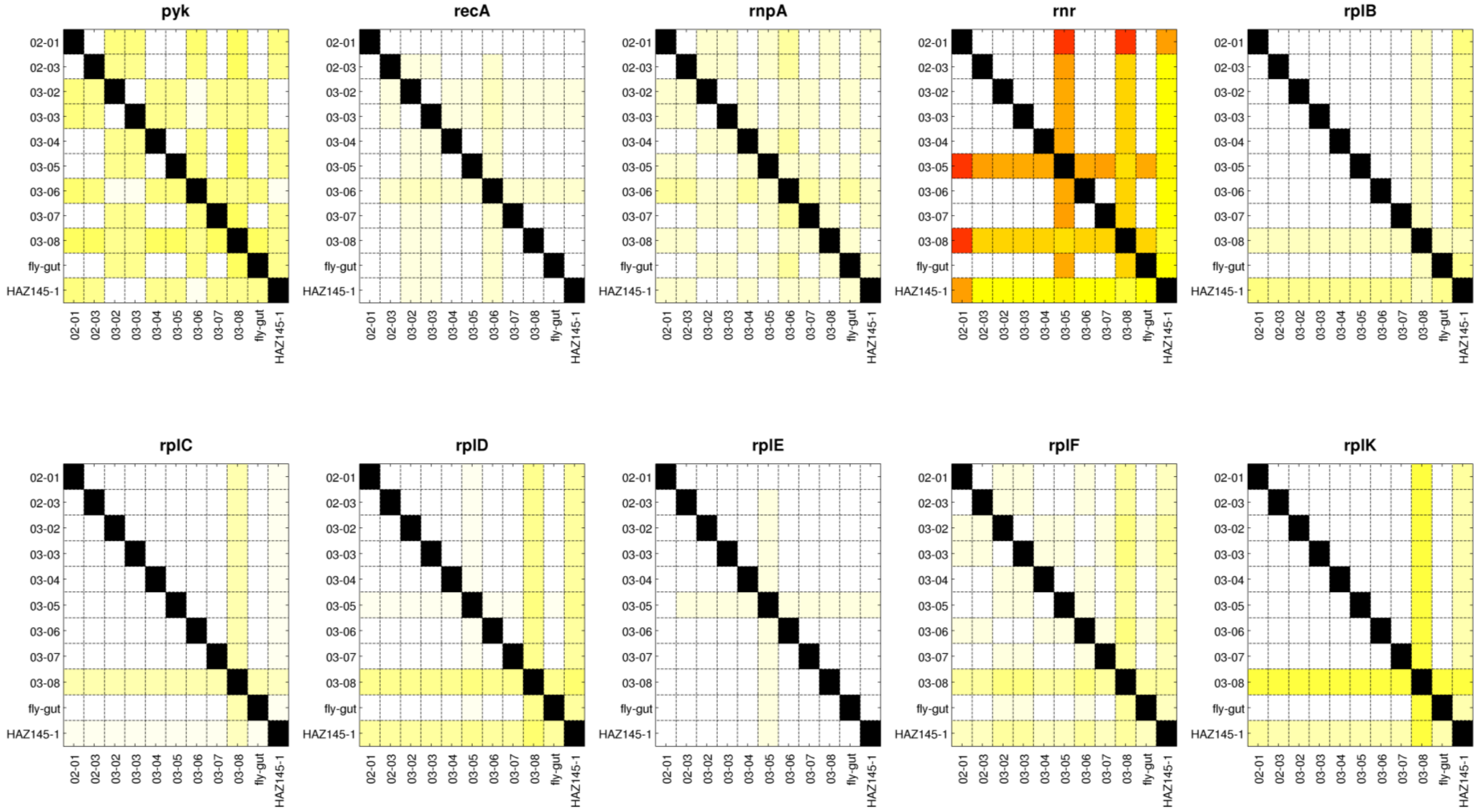

Supplementary Figure 6

Heatmaps illustrating the percentage of identity (Blastn value) between the 10 newly sequenced M. arginini strains and the HAZ145 reference genome. In the figure, genes rplL rplM, rplN, rplO, rplP, rplQ, rplS, rplT, rplU, rplV are represented. A representative set of genes can be found in Figure 4 of the main text

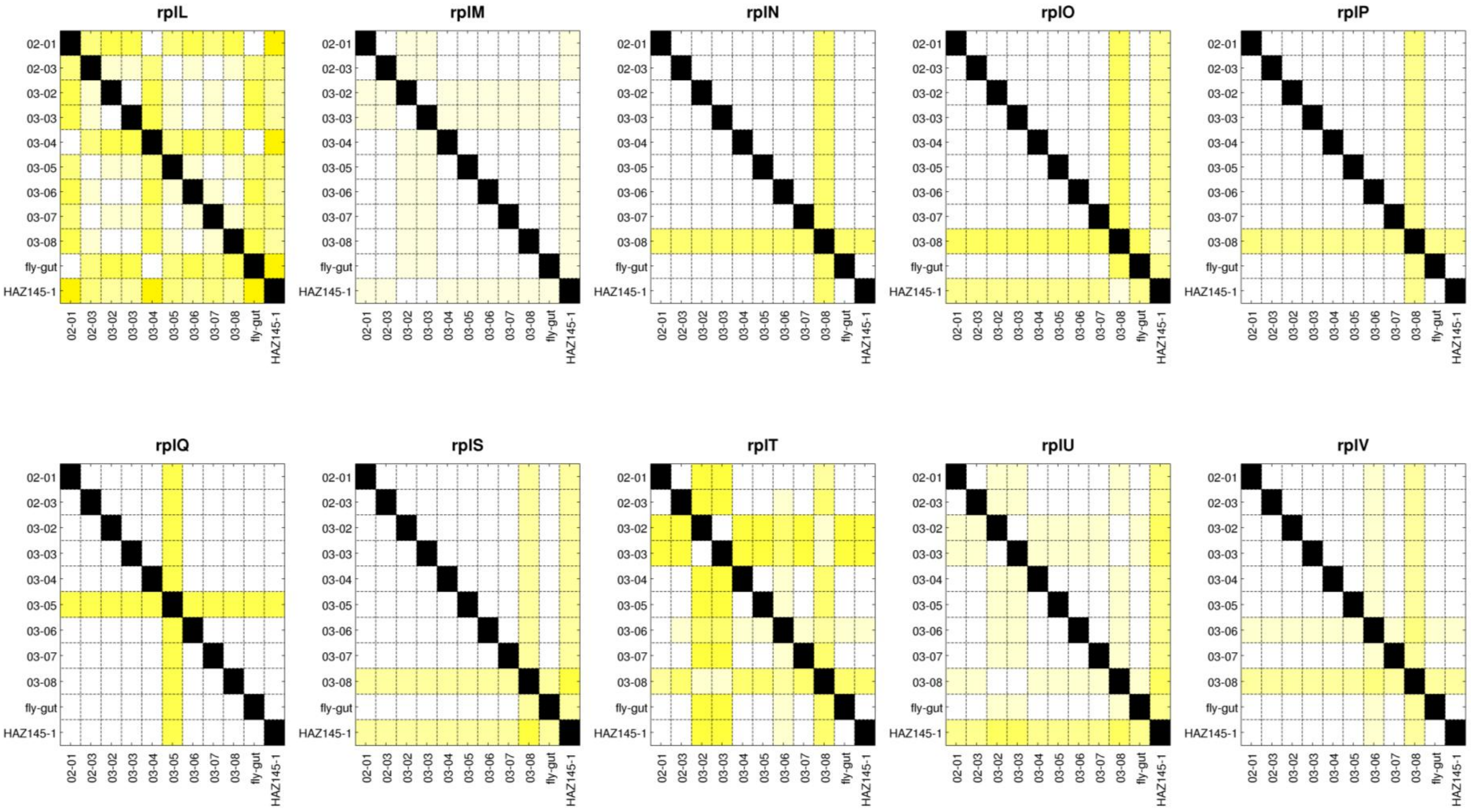

Supplementary Figure 7

Heatmaps illustrating the percentage of identity (Blastn value) between the 10 newly sequenced M. arginini strains and the HAZ145 reference genome. In the figure, genes rpmA, rpmF rpmJ, rpsB, rpsC, rpsD, rpsE, rpsG, rpsH, rpsI are represented. A representative set of genes can be found in Figure 4 of the main text

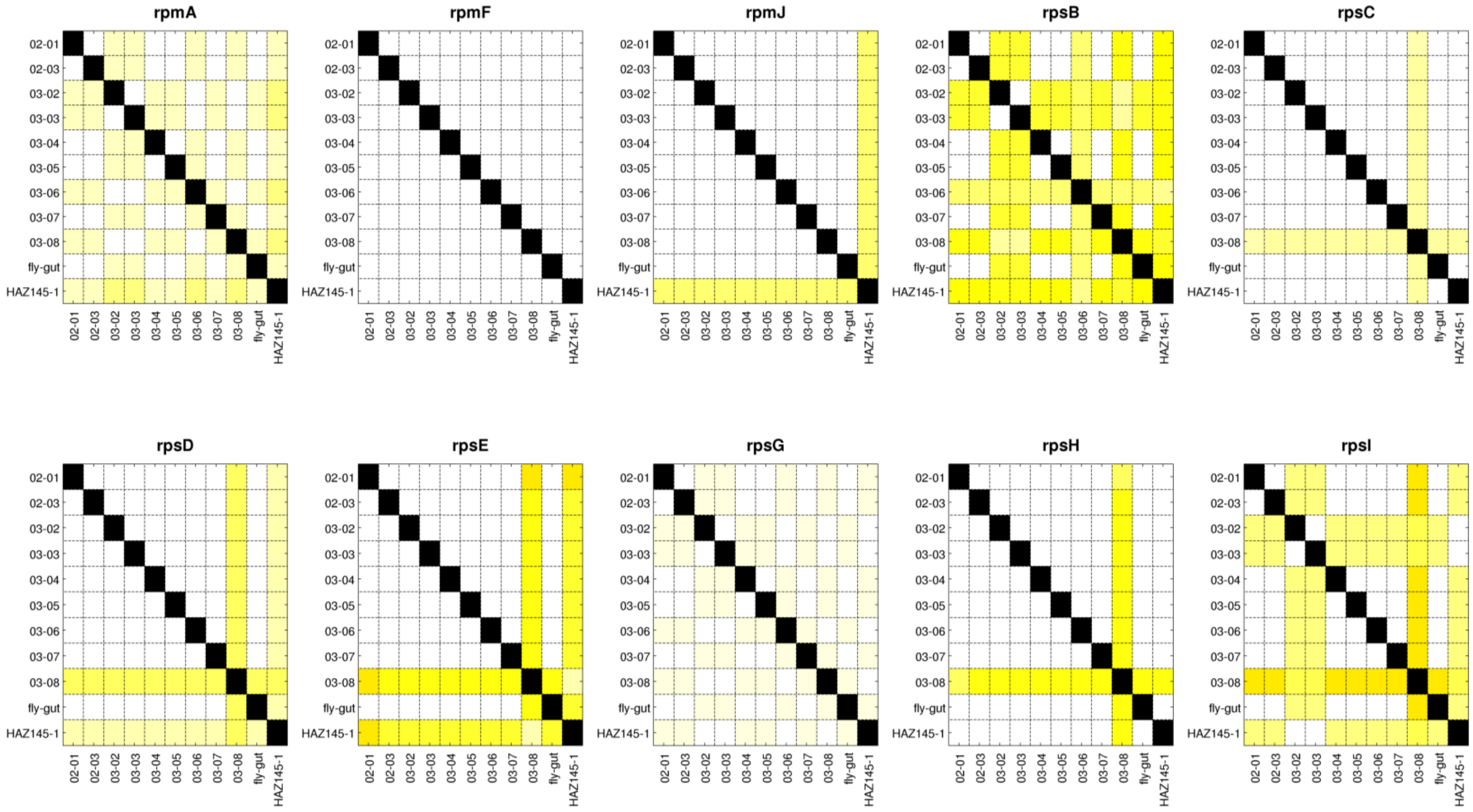

Supplementary Figure 8

Heatmaps illustrating the percentage of identity (Blastn value) between the 10 newly sequenced M. arginini strains and the HAZ145 reference genome. In the figure, genes rpsJ, rpsK, rpsL, rpsM, rpsQ rsgA, rsmD, rsmI, secA, secY are represented. A representative set of genes can be found in Figure 4 of the main text

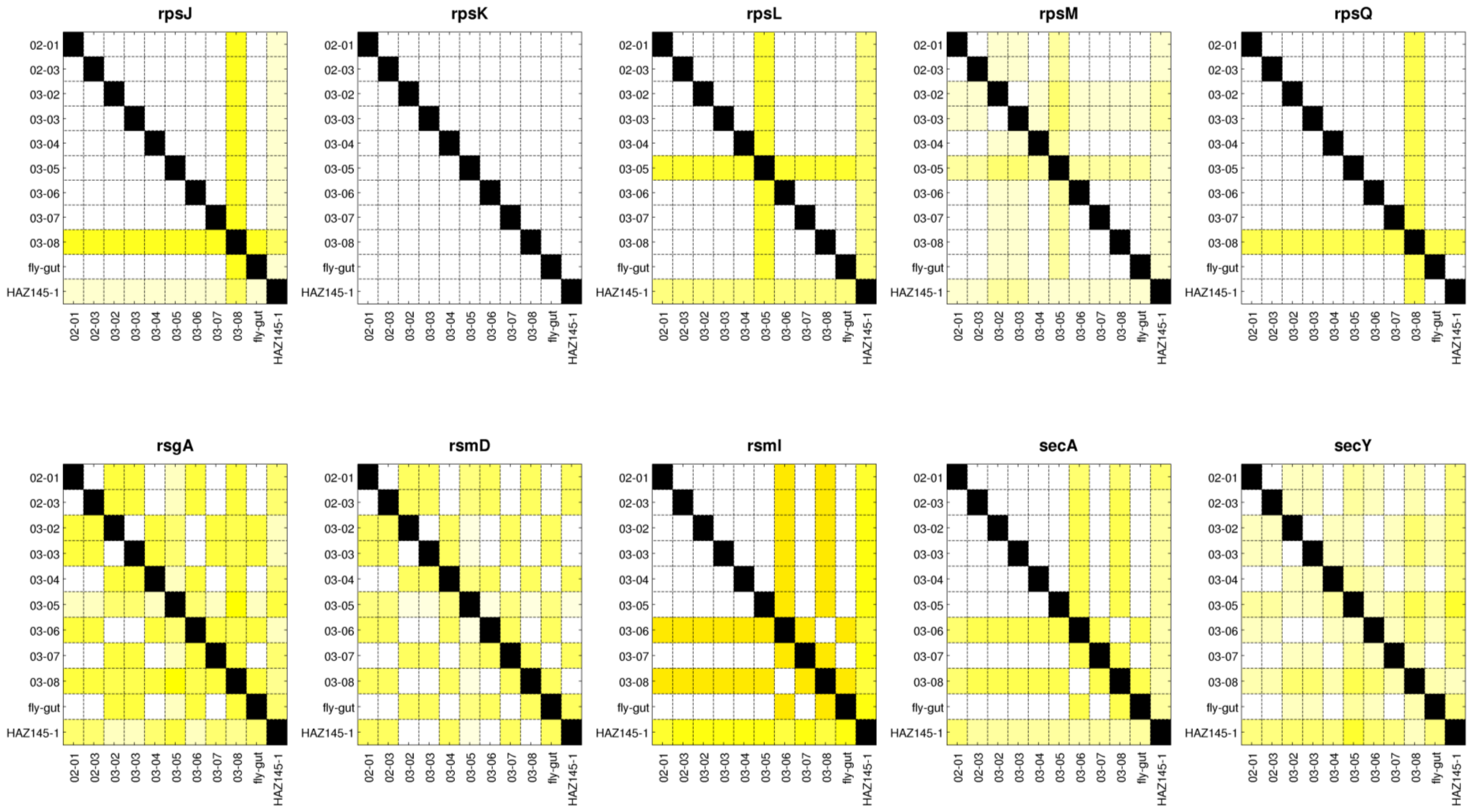

Supplementary Figure 9

Heatmaps illustrating the percentage of identity (Blastn value) between the 10 newly sequenced M. arginini strains and the HAZ145 reference genome. In the figure, genes serS, trmD, truB, tuf, upp, ybeY are represented. A representative set of genes can be found in Figure 4 of the main text

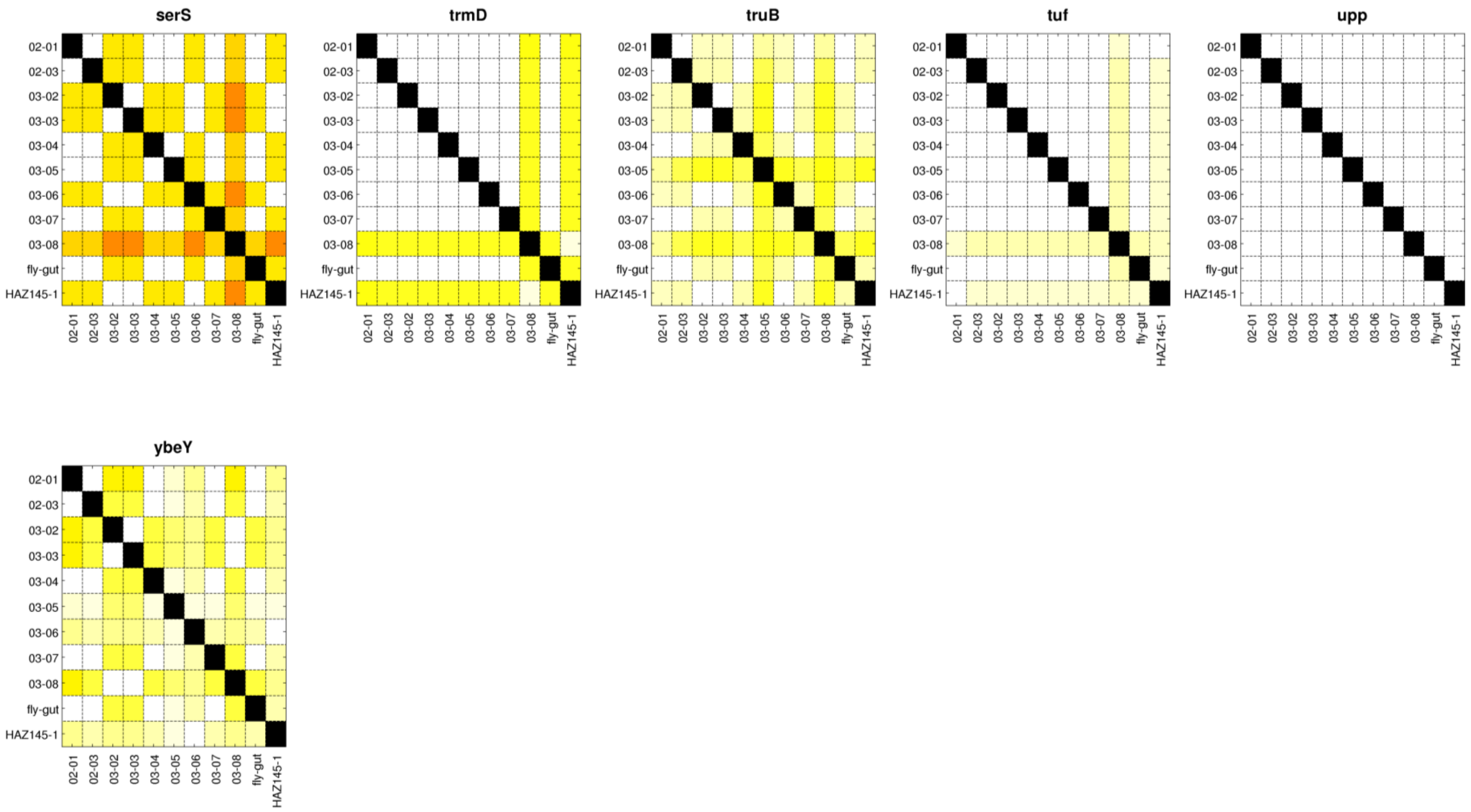

Supplement: Supplemental file 1 — Fig. S1 to S9. Download spectrum.03010-22-s0001.pdf, PDF file, 3.9 MB [file spectrum.03010-22-s0001.pdf]
